# Supplementary material for: Dynamics of nitrogen and active nitrogen components across seasons under varying stand densities in a Larix principis-rupprechtii (Pinaceae) plantation
Source: PeerJ. 2018 Sep 28;6:e5647. doi: 10.7717/peerj.5647 (PMC6166636; doi:10.7717/peerj.5647)
Supplement: Supplemental Information 2 — 35-year-old Larix principis-rupprechtii was the vegetation type of the sample plots above, all the density adjustment work had been done three years before sampling. The data was collected in July 2012, SOC, TN and pH was the average of 5 soil layers 0–50 cm. [file peerj-06-5647-s002.docx]

**Table supplementary 1 Detailed information before density adjustment.** 35-year-old Larix principis-rupprechtii was the vegetation type of the sample plots above, all the density adjustment work had been done three years before sampling. The data was collected in July 2012, SOC, TN and pH was the average of 5 soil layers 0- 50 cm.

| Plot No. | Plot group | Density adjustment | Slope aspect | Slope gradient/° | Mean height/m | Mean DBH/cm | E | N | SOC  g kg^-1^ | TN  g kg^-1^ | pH |
| --- | --- | --- | --- | --- | --- | --- | --- | --- | --- | --- | --- |
|  |  |  |  |  |  |  |  |  |  |  |  |
| 1 | CK1 | 0%(2160 trees·ha^-1^) | N | 30 | 13.39±3.37 | 11.89±3.76 | 112°00′47.1″ | 36°47′05.9″ | 36.51 | 2.79 | 6.17 |
| 5 | CK2 | 0%(2170 trees· ha^-1^) | N | 22 | 16.20±3.08 | 15.01±3.07 | 112°00′44.1″ | 36°47′01.6″ | 31.82 | 2.49 | 6.13 |
| 10 | CK3 | 0%((2188 trees ha^-1^) | N | 23 | 13.97±2.09 | 13.01±3.87 | 112°00′44.4″ | 36°46′58.1″ | 34.33 | 2.47 | 6.12 |
| 6 | LT1 | 15%(1836 trees · ha^-1^) | N | 25 | 13.74±2.69 | 14.61±3.63 | 112°00′43.1″ | 36°47′00.8″ | 37.42 | 2.83 | 6.19 |
| 2 | LT2 | 15%(1839 trees· ha^-1^) | N | 25 | 12.40±3.18 | 14.13±3.82 | 112°00′46.9″ | 36°47′04.6″ | 33.45 | 2.61 | 6.17 |
| 3 | LT3 | 15%(1829 trees· ha^-1^) | N | 24 | 16.28±2.53 | 15.64±3.02 | 112°00′46.2″ | 36°47′03.5″ | 35.61 | 2.53 | 6.09 |
| 9 | MT1 | 35%(1404 trees· ha^-1^) | EN | 23 | 15.04±2.95 | 16.04±3.01 | 112°00′45.2″ | 36°46′59.0″ | 36.66 | 2.63 | 6.11 |
| 11 | MT2 | 35%(1424 trees· ha^-1^) | WN | 22 | 16.41±2.92 | 16.29±3.06 | 112°00′43.5″ | 36°46′58.7″ | 33.85 | 2.80 | 6.17 |
| 7 | MT3 | 35%(1411 trees· ha^-1^) | N | 23 | 16.83±3.83 | 16.33±4.07 | 112°00′41.5″ | 36°46′59.5″ | 35.63 | 2.58 | 6.00 |
| 4 | HT1 | 50%(1080 trees· ha^-1^) | N | 25 | 12.97±2.09 | 15.66±2.23 | 112°00′45.1″ | 36°47′02.5″ | 38.64 | 2.81 | 6.10 |
| 8 | HT2 | 50%(1086 trees· ha^-1^) | N | 26 | 16.60±3.88 | 16.36±4.39 | 112°00′45.2″ | 36°47′02.6″ | 37.32 | 2.87 | 6.17 |
| 12 | HT3 | 50%(1092 trees· ha^-1^) | WN | 22 | 17.21±3.05 | 17.65±3.77 | 112°00′42.8″ | 36°47′57.4″ | 37.32 | 2.87 | 6.17 |
