# Supplementary material for: Dynamics of nitrogen and active nitrogen components across seasons under varying stand densities in a Larix principis-rupprechtii (Pinaceae) plantation
Source: PeerJ. 2018 Sep 28;6:e5647. doi: 10.7717/peerj.5647 (PMC6166636; doi:10.7717/peerj.5647)
Supplement: Supplemental Information 3 — Treatments as in Table 1. Results were collected in 2015 July. NP measns “no presence of.” Importance value = RF + RD + RC, Relative frequency (RF) = (frequency of the specie / the sum of all species frequency) * 100%; Relative density (RD) = (the number of individual specie / the sum of all species number) * 100%; Relative coverage (RC) = (the coverage of an individual specie / the coverage of all plants) * 100%. [file peerj-06-5647-s003.docx]

**Table supplementary 2. Species list for the understory vegetation of different thinning treatments.** Treatments as in Table 1. Results were collected in 2015 July. NP measns “no presence of”. Importance value = RF + RD + RC, Relative frequency (RF) = (frequency of the specie / the sum of all species frequency) *100%; Relative density (RD) = (the number of individual specie / the sum of all species number) *100%; Relative coverage (RC) = (the coverage of an individual specie / the coverage of all plants) *100%.

|  | Family | Genus | Species | Important Value (%) | | | |
| --- | --- | --- | --- | --- | --- | --- | --- |
|  |  |  |  |  |  |  |  |
|  |  |  |  | NTC | LTF | MTF | HTF |
| Herb Layer | Liliaceae | *Hemerocallis* | *Hemerocallis fulva* | NP | 2.48 | 0.86 | NP |
|  |  | *Allium* | *Allium chrysanthum* | NP | NP | 1.57 | NP |
|  |  | *Gagea* | *Gagea pauciflora* | NP | NP | 0.84 | 1.52 |
|  | Labiatae | *Rabdosia* | *Rabdosia japonica* | NP | 1.89 | 1.86 | 0.90 |
|  |  | *Phlomis Linn.* | *Phlomis umbrosa* | NP | 0.62 | NP | 2.40 |
|  | Leguminosae | *Vicia* | *Vicia unijuga* | NP | 0.66 | NP | NP |
|  | Balsaminaceae | *Impatiens* | *Impatiens noli-tangere.* | NP | NP | NP | 6.30 |
|  | Gramineae | *Spodiopogon* | *Spodiopogon sibiricus* | 2.79 | 6.04 | 7.71 | 6.03 |
|  | Saxifragaceae | *Astilbe* | *Astilbe chinensis* | NP | 4.84 | 3.88 | 4.18 |
|  | Compositae | *Gerbera Cass.* | *Gerbera anandria* | NP | NP | 0.47 | 1.98 |
|  |  | *Prenanthes* | *Prenanthes macrophylla* | NP | NP | 0.80 | NP |
|  |  | *Adenocaulon* | *Adenocaulon himalaicum* | 1.13 | 1.22 | 0.77 | NP |
|  |  | *Senecio* | *Senecio nemorensis* | 5.92 | 9.93 | 2.09 | 3.18 |
|  |  | *Aster* | *Aster ageratoides* | 7.19 | 10.43 | 10.59 | 14.34 |
|  |  | *Eclipta* | *Eclipta prostrata* | 28.16 | 10.27 | 15.35 | 14.00 |
|  |  | *Saussurea* | *Saussurea parviflora* | 2.33 | 7.21 | 15.34 | 15.48 |
|  |  |  | *Saussurea sylvatica* | NP | NP | NP | 1.61 |
|  |  | *Dendranthema* | *Dendranthema chanetii* | 6.21 | 4.76 | 8.93 | 2.64 |
|  | Selaginellaceae | *Selaginella* | *Selaginella sinensis* | 1.13 | NP | NP | NP |
|  | Onagraceae | *Epilobium* | *Epilobium angustifolium* | NP | 0.62 | NP | 1.47 |
|  | Geraniaceae | *Geranium* | *Geranium platyanthum* | NP | 1.55 | 1.58 | NP |
|  | Ranunculaceae | *Aquilegia* | *Aquilegia yabeana* | 1.28 | 1.25 | 0.86 | 1.13 |
|  |  | *Actaea* | *Actaea asiatica* | NP | NP | NP | 0.90 |
|  |  | *Aconitum* | *Aconitum carmichaeli* . | NP | NP | NP | 1.08 |
|  |  | *Thalictrum* | *Thalictrum petaloideum* | 17.77 | 11.33 | NP | NP |
|  |  | *Pulsatilla Adans.* | *Pulsatilla chinensis* | NP | NP | 0.60 | NP |
|  | Equisetaceae | *Equisetum* | *Equisetum arvense* | NP | 1.60 | 7.41 | 3.97 |
|  | Rubiaceae | *Galium* | *Galium verum* | NP | NP | 1.61 | NP |
|  |  |  | *Galium aparine* | 1.13 | 2.88 | 2.29 | NP |
|  | Rosaceae | *Sanguisorba* | *Sanguisorba officinalis* | NP | 0.58 | 0.70 | NP |
|  |  | *Filipendula* | *Filipendula palmata* | NP | NP | 1.20 | NP |
|  |  | *Duchesnea* | *Duchesnea indica* | 4.76 | 7.03 | 4.80 | 5.34 |
|  | Umbelliferae | *Angelica L.* | *Angelica dahurica* | NP | 1.12 | 1.09 | 2.73 |
|  | Cyperaceae | *Carex* | *Carex stenophylla* | 13.80 | 7.83 | 4.52 | 5.27 |
|  | Cruciferae | *Arabis* | *Arabis hirsuta.* | 6.38 | 1.64 | 1.94 | 2.58 |
|  | Caryophyllaceae | *Myosoton* | *Myosoton aquaticum* | NP | 0.93 | NP | 0.94 |
|  | Aristolochiaceae | Asarum | *Asarum sieboldi*i | NP | 1.30 | 0.31 | NP |
| Shrub Layer | Caprifoliaceae | *Lonicera* | *Lonicera japonica* | NP | 100 | 55.56 | 46.30 |
|  | Leguminosae | *Cytisus* | *Cytisus scoparius* | NP | NP | 44.44 | NP |
|  | Saxifragaceae | *Ribes* | *Ribes burejense* | NP | NP | NP | 27.78 |
|  | Rosaceae | *Rosa* | *Rosa xanthina* | NP | NP | NP | 25.93 |
